# Nonconsensual neurocorrectives, bypassing, and free action

**DOI:** 10.1007/s11098-021-01740-y

**Published:** 2021-09-26

**Authors:** Gabriel De Marco

**Affiliations:** Oxford Uehiro Centre for Practical Ethics, University of Oxford, Oxford, Oxfordshire UK

**Keywords:** Free action, Manipulation, Bypassing, Neurocorrectives, Neurointerventions

## Abstract

As neuroscience progresses, we will not only gain a better understanding of how our brains work, but also a better understanding of how to modify them, and as a result, our mental states. An important question we are faced with is whether the state could be justified in implementing such methods on criminal offenders, without their consent, for the purposes of rehabilitation and reduction of recidivism; a practice that is already legal in some jurisdictions. By focusing on a prominent type of view of free action, which I call bypassing views, this paper evaluates how such interventions may negatively impact the freedom of their subjects. The paper concludes that there will be a tension between the goals of rehabilitation and reduction of recidivism, on the one hand, and the negative impact such interventions may have on free action, on the other. Other things equal, the better that a particular intervention is at achieving the former, the more likely it is to result in the latter.

## Introduction

As neuroscience progresses, we will not only gain a better understanding of how our brains work, but also a better understanding of how to modify them, and as a result, our mental states. An important question we are faced with, which will become even more relevant as these methods develop, is whether the state could be justified in implementing such methods on offenders in order to aid its goals in rehabilitating offenders and reducing crime rates.^1^ In some jurisdictions, the use of anti-libidinal drugs, intended to reduce the testosterone of sexual offenders, is already legal.^2^ Other potential interventions include, for instance, the use of drugs to increase offenders’ empathy, or brain stimulation to reduce aggressive impulses. Call such methods, intended to improve offender behavior via the modification of brain states, *neurocorrectives*. One important question concerns whether the state could be justified in using such methods without the offender’s consent; whether, that is, it could be justified in making them mandatory. Call such correctives *non-consensual neurocorrectives*, or *NNs*, for short.

Plausibly, the state has some reasons to implement NNs, if it has reasons to attempt to rehabilitate offenders and to reduce the rate of these offenses. Plausibly as well, the state has reasons to *not* use NNs. One might think, for example, that we have reason to avoid the risk of repeating the atrocities found in the problematic history of similar practices (McTernan, 2018; Ryberg, 2020, Chapter 6), or that the state should not employ neurocorrectives because they would express a disrespectful message (Shaw, 2014, 2018a), objectify the individual (Shaw, 2014), infringe some right or other (e.g., to bodily and/or mental integrity) (Bublitz, 2018; Shaw, 2018a, 2018b, 2019), reduce the deterrent effect of criminal justice systems, or violate norms governing medical procedures (Douglas et al., 2013, p. 403).^3^

One important feature of NNs, which may give us some reasons for or against their implementation, is the effect they have on offenders’ freedom.^4^ Some have argued that moral enhancements or moral therapies, some of which may be used as neurocorrectives, may affect freedom by *improving* the features relevant to free agency in some way or other; for example, the subject’s deliberative capacity, or her capacity to recognize reasons (Bomann-Larsen, 2013, p. 69; Caplan, 2006; Douglas, 2013; Douglas et al., 2013; Glannon, 2008, 2011; Lev, 2012; Ryberg, 2012, p. 233; Savulescu & Persson, 2012; Schaefer et al., 2014; Vincent, 2011, 2013, 2014).^5^ This can happen in various ways; e.g., by expanding the set of reasons the agent can recognize, by increasing the perceived strength of moral reasons, or by removing impediments to recognizing or adequately responding to them, such as addictive desires. If freedom can come in degrees, then NNs may *increase* freedom by increasing the extent to which one has the relevant capacities.

However, according to some views of free action, NNs might reduce, or eliminate subjects’ freedom with respect to some actions. These views, and how they apply to NNs, are the topic of this paper. In the next section, I introduce these views and the motivation for holding them. In Sect. 3, I present these views in detail, as well as some of their limitations. In Sect. 4, I discuss how such views might inform the debate on the ethics of NNs.

## Manipulation and bypassing

Consider a pair of cases presented by Mele (2006, pp. 168–169). In the past, Pat occasionally felt guilty about being a mediocre father, and he decided to change this. In order to do so, Pat designed and executed a long-term plan for self-improvement. After years of work, he ended up being the wonderful father that he is today. This process resulted in parental values that are such that it would take Pat a significant amount of time to change them.^6^ Further, they are so strong that, although his faculties for rational control are not impaired, he cannot do otherwise than make certain sacrifices for his children, because he can see so clearly what the situation is. Today, he decided to make a sacrifice and take out a substantial loan to pay for his daughter’s first year at an expensive college.

Now compare Pat to Paul, a mediocre father who has, for many years, reflectively identified with his selfish values. A team of scientists determined what makes Pat such a great father and used that knowledge to make Paul more like Pat. As Paul slept, the scientists implanted much of Pat’s hierarchy of values in Paul and erased competing values. When it comes to his attitudes towards his children, Paul is now very much like Pat, and were he to critically reflect on his own values and priorities, he would conclude the same thing that Pat would of himself. When Paul awakes, he remembers his daughter’s wish to go to an expensive college and experiences a strong desire to take out a loan to help her do that. Paul is surprised by this and wonders why, all of a sudden, he cares so much about his daughter’s welfare and not very much about the new car he wanted. He figures that he has become tired of his selfish ways and he finally sees the importance of a father-daughter relationship, and “[w]hen he carefully reflects on his values, Paul…wholeheartedly embraces the idea of living such a life and the values that support it” (Mele, 2019, p. 65). Later that day, Paul decides to take out a substantial loan to finance his daughter’s first year in college and does so. Due to his new parental values, he could not have done otherwise, in the same sense that Pat could not have done otherwise.

Pat and Paul are identical with respect to various features often considered relevant for free action; e.g., at the time of decision, and shortly before, they are both responsive to reasons, they exercise their rational capacities to deliberate about whether to pay for their daughter’s school, they both have positive second-order attitudes—i.e., attitudes about attitudes—concerning the first-order attitudes that lead them to their decisions, etc. These features of agents are *synchronic*; what happened in the past is irrelevant to whether the agent has them at the time of action, or shortly before. In terms of synchronic properties, the two agents do not differ. However, it seems that Pat freely makes his decision, whereas Paul does not.^7^

What, then, can account for this difference in freedom between Pat and Paul? The main approaches to answering this question suggest that the difference is in *diachronic* features; features which are such that having them will depend, at least partly, on what happened in the past. On a prominent version of this view, part of the difference is the fact that Paul’s action issues from attitudes that were acquired in a way that bypassed his capacities for control over his mental life—e.g., the capacities to critically assess, endorse, and sustain one’s values (Fischer, 2012; Fischer & Ravizza, 1998; Haji & Cuypers, 2008; McKenna, 2016; Mele, 1995, 2006, 2019).^8^ From now on, I simply refer to the process of bypassing these capacities as *bypassing*,^9^ and I refer to views that appeal to bypassing in their explanation of Paul’s lack of freedom as *bypassing views*.^10^^,^^11^

## Bypassing views

A core feature of bypassing views is that they single out attitudes acquired via bypassing in accounting for the difference between typical agents like Pat and manipulated agents like Paul. Consider a simple version of such a view, offering a sufficient condition on lack of freedom for an action:

BS: If action *A* issues from an attitude acquired via bypassing (that the agent neither arranged nor consented to), then the agent does not freely *A*.

Sometimes, bypassing views are interpreted as versions of BS. For instance, of Mele’s view, it is said that:

the mere fact that an agent acquired a certain psychological attitude in a way that bypassed her capacities for rational control is sufficient for denying her responsibility for actions that issue from that attitude (Sharp & Wasserman, 2016, p. 181)^12^

Or, for example, under the supposition that in order to act freely, the action must issue from an authentic attitude:

agents *authentically* possess pro-attitudes acquired through direct interventions that bypass control capacities if, and only if, the agents have arranged for the interventions themselves and foreseen the results (Bublitz & Merkel, 2009, p. 370).^13^^,^^14^

As some, including bypassing theorists, have pointed out, a view like BS would be too simple. We are subject to a variety of influences in our daily lives, and it is quite possible that many of these influence our attitudes via bypassing. A view like BS might then risk deeming too many of our actions unfree, in virtue of the origins of the attitudes they issue from (See Arpaly 2006, p. 115; Bublitz and Merkel 2009, p. 371; Fischer 2012, p. 199; Frankfurt 2002, p. 28; McKenna 2017, pp. 579–580; Mele 1995, p. 157).

On current bypassing views, however, BS is false; the fact that an action issues from attitudes acquired via bypassing (which the agent neither consented to nor arranged) is not sufficient to undermine freedom for that action. In order to undermine freedom for some action, other features will need to be present as well.

One such feature, which bypassing theorists seem to agree on, concerns the significance of the change, or how substantial it is; if the change is fairly insignificant, then it is doubtful that it will undermine our freedom for an action influenced by it. For example, if the effect of the intervention.

is no different in any relevant respect than the way that, for instance, a momentary alteration in attention due to bad digestion might affect someone’s deliberation or subsequent decision, or a quick spike in blood sugar, or an unexpected remark about one’s abusive father…and if all the other control elements are held in place and operate in non-deviant fashion, it is hard to see why we should think that an agent’s freedom or control is impaired (McKenna, 2017, pp. 579–580).

Many of the mundane influences that might affect our attitudes via bypassing will likely be of this sort. On Fischer’s view, the change that occurred via bypassing needs to be substantial enough to make it such that the mechanism leading to action—understood as the process leading to action—is of a different kind than one that the agent has previously taken responsibility for (Fischer & Ravizza, 1998, Chapter 8). Mele suggests that if the change in attitudes resulting from bypassing is not very significant, then the agent may still act freely on the basis of the changed attitudes (Mele, 2019, pp. 35–38), and Haji also claims that significance of change will matter (Haji, 1998, p. 132, 2010, p. 278).

What might determine the significance of the change, or how substantial it is? There are various candidates we might consider, and views can diverge on what they take to be important in answering this question. One set of features may concern the attitudes themselves. For instance, some things that might affect significance may be the number of attitudes changed, the degree of change in individual attitudes, the centrality of these attitudes to the person’s “deep” or “real” self, or whether the changed attitudes are first- or second-order. Another set of features may concern the agent’s abilities. For instance, the significance of the change may be partly a function of the degree to which, or perhaps the ease with which, the agent can resist the changed attitudes; or perhaps the extent to which, or the ease with which, an agent can modify the changed attitudes.^15^ Put simply, the significance of the change may depend on the breadth and depth of the change, relative to the action at hand; and this can be understood in terms of either the attitudes affected, or the agent’s abilities (or both).

Bypassing views often mention other relevant features, which may or may not be understood as a dimension of the significance of the change. For example, on McKenna’s view, attitudes acquired via bypassing can undermine freedom only if they are *unsheddable*: only if they are such that, in normal contexts of practical deliberation, the agent can neither eliminate nor weaken them by the time of action (McKenna, 2016, pp. 88–89). Further, in order for an unsheddable attitude acquired via bypassing to undermine freedom, the agent needs to have lacked “the opportunity to critically assess, endorse, and sustain [the attitude] from abilities that she possessed” (McKenna, 2016, p. 97). Fischer similarly states that one must have “a reasonable and fair opportunity to filter a new [attitude] through my character and practical reasoning” (Fischer, 2012, p. 199).^16^ We will return to this notion of opportunity shortly.

Mele’s view is of a different form than the others, since he does not intend to provide a necessary condition on free action that accounts for all problematic cases of manipulated agents. His most recent condition is intended to help explain why agents like Paul, in particular, are not free or responsible for the relevant actions:

*NFMR.* An agent does not freely *A* and is not morally responsible for *A-*ing if the following is true:

1. for years... his system of values was such as to preclude his acquiring even a desire to perform an action of type *A*, much less an intention to perform an action of that type;
2. he was morally responsible for having a long-standing system of values with that property^17^;
3. by means of very recent [bypassing] to which he did not consent and for which he is not morally responsible, his system of values was suddenly and radically transformed in such a way as to render *A*-ing attractive to him during *t*; and
4. the transformation ensures either
  - that although he is able during *t* intentionally to do otherwise than *A* during *t*, the only values that contribute to that ability are products of very recent [bypassing] and are radically unlike any of his erased values (in content or strength) or
  - that, owing to his new values, he has at least a Luther-style inability during *t* intentionally to do otherwise than *A* during *t*. (Mele, 2019, pp. 66–67)^18^

Conjuncts 1 and 3 specify the sort of change that needs to happen via bypassing in order for the view to say that the agent did not freely act. The change is quite stark, though this is mainly because the condition is intended to capture cases like Paul’s, which involve a radical change. Conjunct 4 mentions Luther-style inability, in reference to Dennett’s discussion of the phrase famously attributed to Martin Luther: “Here I stand, I can do no other” (Mele, 2019, pp. 62–64). The most concise characterization is expressed by Dennett when he states that: “when I say I cannot do otherwise I mean I cannot because I see so clearly what the situation is and because my rational control faculty is *not* impaired” (Dennett, 1984, p. 133). Notably, this sense of (in)ability is concerned with doing otherwise in relevantly similar circumstances.

All four of these conjuncts are true of Paul’s action, which also fails to meet necessary conditions offered by the other views. Pat’s action, on the other hand, seems to meet all of them; his parental values were ones that he developed over the years and were not acquired via bypassing. Thus, these views can account for the claim that Pat acts freely yet Paul does not. However, one might think that although Paul is not free for some actions issuing from attitudes acquired via bypassing, he can eventually freely perform such actions in the future. Perhaps over time, and given the right opportunities to evaluate his parental values, he will be able to freely act on them.^19^ Since the debate concerning bypassing views has mainly focused on actions manipulated agents perform shortly after the manipulation, explaining how they might regain freedom with respect to similar actions in the long term has not been explored in depth.

On McKenna and Fischer’s views, a lack of opportunity to evaluate the implanted states is part of what explains why the agent did not act freely. This initially suggests a simple solution: once the agent has this opportunity, she can regain freedom for actions issuing from the relevant sorts of states. Yet, for this response to work, we will need to know more about the nature of this opportunity. Recall that the morning after the manipulation, Paul has a bit of time to consider his new values, and does so; importantly, he does so before deciding to take out a loan for his daughter’s education. If this opportunity to consider the implanted attitudes were sufficient for him to regain freedom for actions issuing from them, then his decision to take out the loan would have been free, and the account would be unable to explain why it was *not* free. The difficulty stems, in part, from the fact that his evaluation was wholly guided by further implanted attitudes.^20^ We can call cases with this feature the *hard cases* of regaining freedom.

To date, no bypassing theorist has offered a fully developed view that solves these hard cases. Perhaps, following Fischer, one could claim that the opportunity that Paul had before making his unfree decision was not a *reasonable and fair* opportunity; though a satisfying version of this response would involve saying more about what *would* make an opportunity reasonable and fair.^21^ Another way of approaching the problem would be to offer a story of how this might happen, as Mele does. To adapt that story to the case of Paul, consider Paul a year later. He still has strong parental values, and in some situations, cannot do otherwise than act on them, in the sense specified above. Further suppose that Paul had, for a long time, valued his happiness above all; a value that was unaffected by the manipulation, and has persisted since. In the past year, he has come to learn that what makes his life happiest is the welfare of his children, and his relationship with them. So, he has come to “reflectively embrace and identify with the values at issue—products of manipulation—as part of a package that supports what [he] always valued most” (Mele, 2020, p. 3149).

It is important to note, however, that not *all* cases will be as difficult as Paul’s. There are easier cases, in which we would not expect the evaluation of the implanted states to be wholly guided, or perhaps even significantly influenced, by further implanted states. Consider the case of Judith, based on one introduced by Fischer and Ravizza (1998, pp. 232–233). Without her knowledge, manipulators implanted in Judith a very strong desire to punch her friend Jane in the face; a desire which would be difficult to resist. When Judith sees Jane the next day, she acts on this desire. One might think that she does not freely do so; for our purposes, we can suppose this is right.

Unlike Paul, Judith has recently acquired, via bypassing, only this very strong desire; the manipulators do not implant any second-order attitudes. Thus, were Judith to reflect on this implanted desire, she may well fail to endorse it; the evaluation would not be supported by further attitudes recently acquired via bypassing. To use some terminology that is common in these debates, we can say that Judith’s manipulation is *local*, whereas Paul’s is more *global*. Agents like Paul, who are subject to more global manipulation, are likely to be hard cases; whereas agents like Judith, who are subject to more local manipulation, will not be.

How might agents like Judith come to freely perform actions issuing from the attitudes they acquired via bypassing? Here I offer some suggestions, partly inspired by Fischer’s own discussion of such cases (Fischer, 2012, Chapter 11; Fischer & Ravizza, 1998, p. 235).^22^ Suppose that Judith has now had the opportunity to evaluate the desire. If, after considering and evaluating the desire, she judges that she does not want to change it significantly, then actions issuing from it can be free. If, however, she judges that she should change or eliminate it, then insofar as she retains this disposition towards the implanted desire, she does not act freely from it if (a) she has not had the opportunity to modify or eliminate it, and (b) she has not had the opportunity to learn how to reliably resist it in the contexts like the current one.^23^

With this general characterization of bypassing views in place, we can now engage with the question of whether and how, on these views, NNs would negatively affect freedom for some actions.^24^

## NNs and bypassing

The main motivation for NNs is that they modify offender behavior, thereby helping to rehabilitate offenders and reduce recidivism. These intended effects work by modifying subjects’ brain states and corresponding mental states. Since NNs are non-consensual, and do not engage with the subjects’ capacities for control over their mental lives, their effects on subjects’ mental states occur via bypassing. Although they share this feature, neurocorrectives are quite diverse in their methods, intended effects, and degrees of efficacy. What bypassing views have to say about specific sorts of NNs will depend on the particular features of the neurocorrective in question, and on the nature of the subject of the NN. My focus will be on what general claims we can make about the effects that NNs might have on subjects’ freedom.

Yet we can still narrow down the subject matter somewhat. As previously mentioned, it is plausible that we have some reasons not to implement NNs. The reasons in favor of implementing them, reasons which might outweigh the reasons against, concern the possibility of rehabilitation of offenders and reducing recidivism. Neurocorrectives which do not help in achieving these ends will likely not be good candidates for NNs, since there will not be reasons in favor of their implementation that plausibly outweigh the reasons against. Thus, such neurocorrectives will not be of great concern for our purposes here.

This section is divided into three parts. First, I consider questions relating to whether NNs might undermine an agent’s freedom for some actions. Second, I discuss how subjects whose freedom for some actions has been undermined may regain freedom for these sorts of actions. Finally, I consider the possibility that freedom comes in degrees, and what implications this has for our main question.

### Undermining freedom

One important feature of NNs to keep in mind is that, as they are typically described, they are targeted. That is, they are intended to work via the production of a specific set of attitudes—say, those arising from increased empathy—or the elimination of a specific set of attitudes—say, the sexual urges meant to be eliminated by anti-libidinal neurocorrectives. An NN that is relatively targeted is unlikely to undermine freedom for *all* of a subject’s actions. The attitudes that were acquired or significantly changed via bypassing will not be relevant to all contexts of deliberation, and the subject will likely perform a variety of actions that do not issue from such states. Even if, for example, an anti-libidinal drug significantly modifies some of the subject’s values and desires, the subject may still freely make a decision about whether to pay a visit to his ailing mother. If NNs undermine freedom for actions, it will likely only be with respect to *some* actions.

One feature that bypassing views agree on is that, in order to undermine freedom for some action, the change that occurs via bypassing should be significant, where significance can be roughly understood as a measure of the breadth and depth of the change. The more significant the change that a particular NN produces, the more likely it is to meet any particular significance criterion. Consequently, the more significant the change produced, the more likely that the NN will undermine freedom for some actions. How likely this is, I suggest, will depend on the state’s policy, and its expectations.

First, the goal of rehabilitating offenders and reducing recidivism will be importantly related to the significance of the change. If the most that a particular NN does to any individual is produce changes that are not relevantly different from how “a momentary alteration in attention due to bad digestion might affect someone’s deliberation or subsequent decision, or a quick spike in blood sugar” (McKenna, 2017, p. 579), then it is doubtful that the NN will be of much help in rehabilitating offenders or in reducing recidivism. NNs intended to achieve these goals would likely need to be more significant, and thus risk undermining subjects’ freedom with respect to some actions.

Further, the significance of the change required to achieve these goals will also be related to the nature of the offenders that the NN is intended to change. Rehabilitating some subjects may not call for a very significant change in attitudes, whereas rehabilitating others may require a very significant change. Given that NNs are non-consensual and often perceived to be quite invasive, they are likely to be reserved for perpetrators of particularly serious offenses, and/or for repeat offenders. Suppose that the state restricted the implementation of NNs to such offenders.^25^ Such a policy would seem to select for subjects whose rehabilitation calls for a fairly significant change and thus, for NNs that produce one.

These points apply to effects of NNs that help to achieve the goals of the state. However, as is sometimes the case with biological and neurological interventions, NNs may in some instances result in unintended side-effects. In relation to this, it is important to note that even if an effect is unintended, it can still be a change that occurs via bypassing. Consequently, such side-effects will need to be taken into account in the same way we would take the intended effects into account.^26^

Side-effects resulting in changes via bypassing can widen the range of actions for which freedom is threatened. Suppose, for instance, that an empathy increasing drug has a side-effect resulting in the subject gaining a preference for comedy movies. This might then threaten her freedom for cases in which she is making a choice between two such movies, if the change is significant enough.

However, gauging the likelihood that an NN will undermine freedom in this way will be more difficult. As I suggested above, one of the important factors in selecting which neurocorrectives to administer as NNs will be the significance of the intended effect; in order to aid rehabilitation and the reduction of recidivism, the neurocorrective will likely need to produce significant intended effects.^27^ This does not give us much guidance in relation to side-effects, since significant side-effects will not provide a reason in favor of implementing the NN; in fact, they would seem to provide a reason against. For similar reasons, we would not get much guidance on this from the point concerning subject-selection.

So far, I have considered some of the relevant factors in determining whether an NN will undermine offenders’ freedom for at least some actions, including the significance of both intended and unintended effects, and the nature of the state’s policy, including which sorts of offenders are selected as subjects. However, these are just general points; particular NNs will likely affect different people in different ways, and these effects are unlikely to be uniform.

In cases where NNs have an effect that meets the significance criterion, we seem to have a pro tanto reason to refrain from using them. This is not to suggest that this is the only reason to refrain from using an NN in a particular case, nor is it to suggest that this reason overrides all other reasons in favor of implementing it; as suggested earlier, there are many considerations to take into account. Yet one might object to the claim that we would have such a pro tanto reason. One might think that if an NN eliminates offenders’ freedom to commit serious offenses – say, to murder or rape – this does not give us *any* reason to refrain from implementing them, since this is not a valuable form of freedom. Thus, even if NNs undermine subjects’ freedom with respect to some actions, this does not count against their implementation.^28^

In response, I wish to suggest that the issue here is not with offenders’ freedom to commit serious offenses, but rather with whether they freely perform other actions. The fact that an NN might eliminate, or perhaps merely mitigate, freedom with respect to *these* actions provides us with a pro tanto reason to refrain from their use, and this does not imply that the freedom to commit serious offenses is valuable.

To see why, consider the case of Paul again, the mediocre father whose evaluative scheme was radically modified and as a result, decides to take out a substantial loan to finance his daughter’s education. He does not freely decide to do so, and bypassing views can account for this. However, note that the claim here is not that he does not freely decide to take out the loan *because* he could not have done something else. Pat, the unmanipulated father, also has a Luther-style inability to do otherwise, yet this does not mean that he does not freely decide to take out the loan.

A similar point can be made concerning the claim that NNs undermine freedom for some actions. Consider a couple of cases. First, suppose that rather than implementing the neurointervention *non*consensually, the state implements the neurointervention only after discussions with the offender reveal that he wants to take it, and the offender gives valid consent to the intervention.^29^ Second, suppose that, after much careful research, we discover a new pedagogical technique that makes it possible to rehabilitate some of these offenders in ways previously thought unimaginable. Further suppose that this technique robustly interacts with the agent’s capacities for control over his or her mental life.^30^

In both of these cases, the subjects may undergo changes as significant as those experienced by the subject of an NN. Further, they may lack the freedom to commit serious offenses, at least to the same extent as a subject of an NN. Yet, although bypassing views do not imply that the subjects fail to act freely in these two cases, they may for the subject of an NN. This difference is not explained by a difference in terms of their freedom to commit serious offenses, since there is no such difference. Thus, it is not clear how the supposition that we have a pro tanto reason to refrain from using NNs, in virtue of the fact that they would result in some subjects not acting freely in some cases, implies that the freedom to commit serious offenses is of any value.

### Regaining freedom

If an NN undermines freedom for some actions, then we would seem to have some reason not to implement it. Yet whether or not agents can regain freedom for these actions, and how easy it would be to do so, may either affect the strength of this reason, or provide us with further reasons against the implementation of NNs. Prima facie, at least, it would seem that the longer the period of time that an NN negatively affects freedom, the stronger the reason(s) we have against its implementation. As suggested above, there is no clear answer to the hard problem of regaining freedom. Until such an account is provided, we may need to settle for some uncertainty with regard to this question.

This is one place in which the targeted nature of NNs may make a difference. If an NN is fairly targeted, and does not result in changes that would significantly affect the agent’s evaluation of the changes that occurred via bypassing, then we may get something akin to one of the easy cases of regaining freedom. Suppose, for instance, that a subject of an empathy-increasing NN is now better at recognizing when her actions may harm other individuals. Further suppose that she already believed that she should avoid harm to others, though she did not always recognize that some of her behavior did harm to others. In such a case, the subject may end up identifying with, and endorsing, the increased empathy on the basis of previously held attitudes; and this might be a relatively quick process. This is a straightforward case of coming to be free with regard to actions issuing from these attitudes.

However, it is doubtful that all cases of NNs will have such happy results. Suppose that a subject of an empathy-increasing NN did not believe that one should put much weight on the suffering of others, or suppose that the subject of an aggression-lowering NN took pride in his aggressive impulses, thinking that they were appropriate. In such cases, and supposing that the NN eliminated the agents’ freedom for some actions, regaining freedom may be quite difficult. Such cases may be more like a version of the Judith case in which she does not want the strong desire to punch her friend in the face that was implanted in her.

Above, I suggested that in a case like this one, in which the agent determines that she wants to change or eliminate the attitude on the basis of previously held attitudes, then insofar as she retains this disposition towards the implanted or changed attitude, actions issuing from it would not be free if a) she has not had the opportunity to modify or eliminate it, and b) she has not had the opportunity to learn how to reliably resist it in the contexts like the current one. If the NN is targeted enough to make this an easy case of regaining freedom, and say, it is not difficult for the agent to modify the attitude, or learn to resist them reliably, then regaining freedom for actions that issue from these attitudes may again be somewhat straightforward.

Yet, here again we encounter an important tension between the goals of implementing NNs, and what would need to happen in order for the agent to act freely from such attitudes. Insofar as the state’s goal in implementing NNs is to rehabilitate offenders and reduce recidivism, they will have an interest in making it difficult for subjects of NNs to have either of the opportunities just mentioned. If doing so were easy, then it would be easy for offenders to significantly thwart the state’s goals, and would likely lead to a much less effective policy of NNs. Assuming that the state would want to prevent this, they would have some reason to make it difficult to have either of these opportunities. Perhaps this could be done by a single implementation of a powerful, and perhaps long-lasting, neurocorrective; or perhaps it could simply be done by a continual administration of the NN. For instance, if the NN is a drug, it may be administered periodically, and a re-administration may undermine much of the work done by an offender, or perhaps rejuvenate the NN’s effect.

It is important to note, however, that such a tension need not always be present, and this is because the subject’s disposition towards the changed attitudes can change over time. Thus, even if a subject of an NN is initially opposed to the change, and assuming that it was significant enough to eliminate freedom with respect to actions resulting from it, she may still come to be free with respect to actions issuing from the implanted attitudes without having either the opportunity to modify or eliminate the attitudes, nor the opportunity to learn to reliably resist it in similar contexts of practical deliberation. This can happen, for example, if the subject ends up identifying with or endorsing the attitudes in a way that is not wholly, or perhaps significantly, guided by further implanted attitudes. That is, she may re-evaluate the attitudes and come to be free with respect actions issuing from them if the case changes into one like the first sort of easy case discussed above. Supposing that this is possible, the state may then have reason to help the subject regain freedom in this way. Suppose, for instance, that there is a course of therapy that engages with the subject’s capacities for control over her mental life, and can help her come to identify with and endorse the implanted attitudes. Offering this therapy to the subject can then help her regain freedom with respect to the relevant actions in a way that is consistent with the state’s goals of rehabilitating offenders and reducing recidivism.^31^

### Degrees of freedom

Finally, one further consideration to take into account is the possibility of degrees of freedom. One might think that agents can be more or less free with respect to some action or other, and that the degree to which one is free is a function of the extent to which one exemplifies freedom-relevant features; e.g., how much control an agent has over some action, or how reasons-responsive the agent was at the time of deliberation and/or action.^32^ Bypassing views, intended to explain why agents like Paul do *not* act freely, offer conditions on when bypassing can *fully* undermine freedom. However, the features picked out by bypassing views can come in degrees as well. For instance, the change effected via bypassing can be more or less significant, and one might have more or less of an opportunity to evaluate and modify attitudes acquired via bypassing. This has important implications.

First, this suggests that an NN can negatively impact freedom even if it does not fully undermine it with respect to some actions, in virtue of merely reducing freedom with respect to some actions. In such cases, we may still have a reason against the implementation of NNs. Moreover, the threshold that NNs need to meet in order to mitigate freedom will presumably be lower than the threshold they would need to meet in order to eliminate it; changes occurring via bypassing that are not significant enough to fully undermine freedom may still be significant enough to reduce it. Thus, it is even more likely that NNs will have a negative impact on freedom.

Second, recall that some have argued that NNs, or enhancements more generally, might *increase* an agent’s freedom in virtue of improving freedom-relevant features. The features that would be improved according to these arguments are synchronic features; e.g., reasons-responsiveness, or control at the time of action (or shortly before). These are the sorts of features that Pat and Paul can have to the same extent, even though Pat freely decides and Paul does not. This positive effect on synchronic freedom-relevant features is consistent with the negative effect on the diachronic features that bypassing views focus on. What should we say about these cases?

Since bypassing views offer a necessary condition on free action, failing to meet it with respect to some action implies that the agent does not act freely, regardless of whether the NN improved some synchronic freedom-relevant features. Suppose, for instance, that all four conjuncts of *NFMR*, the most extreme of the bypassing conditions, are true of a subject’s action. In such a case, the subject would not act freely; regardless of whether the NN improved the subject’s synchronic freedom-relevant conditions.^33^

However, a different sort of case will be much more difficult to assess. Suppose that, although an NN does not fully undermine an agent’s freedom with respect to some action, it does have significant effects, and would typically be taken to mitigate freedom with respect to some actions. But, suppose also that the NN results in an improved capacity to recognize and respond to reasons. In such a case, determining the NN’s overall effect on the agent’s freedom with respect to some actions will be a difficult matter since presumably, the extent to which an action is free is at least partly a function of both the agent’s synchronic freedom-relevant features, as well as the features identified in bypassing views.

This is further complicated by the fact that a necessary condition on free action, as is proposed by bypassing theorists, does not give us a *full* account of free action; importantly, it does not clearly commit one to claims about which synchronic features are freedom-relevant features. For instance, theorists might disagree on whether, in order to act freely, one merely needs responsiveness to reasons, or whether one needs, further, responsiveness to *moral* reasons,^34^ or, they might disagree on whether one also needs the ability to do otherwise at, or shortly before, the time of action.^35^ Thus, even among those who adopt bypassing views, there is room for disagreement about when, precisely, an NN would improve synchronic freedom-relevant features.

## Concluding thoughts

Bypassing views can help to shed light on the ways in which NNs might negatively affect offenders’ freedom with respect to some actions, and thus help to inform the debate on whether NNs could be justified. I have, however, not argued that NNs always do, or always will, undermine freedom. The neurocorrectives that might be implemented as NNs will be of different sorts, and some types of NN will likely have much more significant effects than others. Moreover, even holding the type of NN fixed, we can have varied effects; a particular type of NN may have quite significant effects on some subjects, no effects on others, and for others still, it may be something in between. Determining whether a particular type of NN affects some individual’s freedom with respect to some actions will require a more detailed analysis. A general conclusion on the impact NNs may have on free action is therefore difficult to come by.

However, the discussion above has shed light on tensions between the reasons a state may have in favor of implementing NNs – that they would help with the goals of rehabilitation and reduction of recidivism – and some reasons the state may have against implementing NNs – that they could negatively impact freedom of action.^36^ Given its goals, the state will have more reason to implement an NN that has significant intended effects. Yet, other things equal, the more significant these effects, the more likely the NN is to negatively impact freedom with respect to some actions, either by eliminating that freedom, or merely by mitigating it. Further, the state will likely have an interest in making the effects of the NN long-lasting. This, as we saw, can get in the way of the subjects regaining freedom for some actions; at least in those cases in which the subject does not initially approve of the new attitudes, and the state is not able to help the subject come to see them in a new light.

## Footnotes

1 For an overview of current and near-term future possibilities, see Ryberg (2020, Chapter 1).

2 For an overview of their use and legality in various jurisdictions, see Forsberg (2018).

3 See Vincent (2014, pp. 27–8) for a summary of the debate, Birks and Douglas (2018) for an anthology on the topic, and Ryberg (2020) for a book-length treatment.

4 I will not be considering John Harris’s objection to moral enhancement more broadly, which roughly states that such enhancement (or in this case, neurocorrectives) could interfere with the agent’s freedom by eliminating the “freedom to fall” (Harris, 2016). For responses, see DeGrazia (2014), Douglas (2014), Persson and Savulescu (2016a, 2016b), Pugh (2019), Ryberg (2020, pp. 61–8) and Savulescu and Persson (2012).

5 Vincent focuses on responsibility, and explicitly claims that she is not broaching the free will question (Vincent, 2014, p. 33, n. 13). However, some of the capacities she is concerned with are also relevant to free action.

6 I follow Mele (1995, p. 116) and McKenna (2016, p. 88) in their understanding of “*S* values *X*”: “*S* at least *thinly values X* at a time if and only if at that time *S* both has a positive motivational attitude toward *X* and believes *X* to be good”.

7 There are two worries one might have about the case of Paul that I will not engage with. First, one might worry that post-manipulation Paul is not personally identical to pre-manipulation Paul. For responses, see Haji and Cuypers (2001, pp. 222–233, 2008, p. 225, n. 4) and Mele (1995, p. 175, n. 22). Second, one might think that a possible explanation of why Paul does not act freely is that he cannot do otherwise, in one’s preferred understanding of this ability, and this action cannot be traced back to some previous instance in which he did have the relevant ability. There are similar cases of manipulated agents in which they have the ability to do otherwise in one of the strictest senses available (Cyr, 2016; Haji & Cuypers, 2001; King, 2013; McKenna, 2016, p. 88; Mele, 2019, pp. 124–126; Tognazzini, 2014).

8 Although I call these views of free action, Haji and Cuypers’s view is an exception. They offer an authenticity condition on moral responsibility.

9 Some work in experimental philosophy aims to evaluate what is sometimes called the “bypass hypothesis”, according to which “people take [free will and] responsibility to be undermined by determinism because they take determinism to imply that the agent’s beliefs, desires, and decisions are *bypassed*, playing no role in bringing about or determining the agent’s actions” (Björnsson, 2015, p. 96) [see also (Andow & Cova, 2016; Feltz & Millan, 2015; Murray & Nahmias, 2014; Nahmias et al., 2007)]. In this context, “bypassing” is understood as a global phenomenon that concerns all of an agent’s behaviors, and all of their mental states and capacities. As I use the term, however, it simply concerns whether a *particular* change in attitudes occurred in a way that failed to engage with the agent’s capacities for control over their mental life. The fact that an agent’s capacities were bypassed in the production of such a change does not imply that the agent’s mental states, deliberations, etc. play no role in the production of their actions; e.g., Paul’s decision was influenced by the implanted mental states. Thanks to an anonymous reviewer for pointing out the need for clarification.

10 Sometimes these are called views of authenticity, yet I mostly refrain from using the term. The term is ambiguous, often employed in different contexts and to solve different problems (Garcia, 2015; Newman and Smith, 2016). Of the four main bypassing views, only Haji and Cuypers use the term “authenticity.” Mele has in the past (Mele, 1995), yet has dropped all reference to it in more recent work (Mele, 2006, 2019).

11 There is another set of views that do not appeal to bypassing in order to explain why manipulated agents are not free and/or responsible. Rather, they appeal to the presence of the manipulator’s intention (Barnes, 2015; Yaffe, 2003), or to the causal source of the agent’s action, which in these cases, is the manipulator’s intentional action (Deery & Nahmias, 2017). Which type of view is preferable, or whether we need to choose between them, is an open question. This paper will focus on bypassing views.

12 For a criticism of bypassing views relying on a similar interpretation, see Bublitz and Merkel (2013, pp. 354, and 354 n. 24). Here, Bublitz and Merkel seem to be conflating Fischer and Ravizza’s notion of guidance control with a notion of competence.

13 Perhaps Bublitz and Merkel mistakenly attribute this view to Mele because they quote him as stating that “[Sometimes] agents come to possess pro-attitudes in ways that *bypass* their control capacities over their mental lives…Bypassing is sufficient for compulsion…provided that the bypassing was not itself arranged or performed by the manipulated person” (Bublitz & Merkel, 2009, p. 370) [the same quote appears in Bublitz 2011, p. 111]. The phrase in the middle, “Bypassing is sufficient for compulsion” does not appear anywhere in Mele’s work. Mele explicitly rejects BS (Mele, 1995, pp. 148–149, 158–159, 2006, p. 167), and distinguishes between forms of compulsion (Mele, 1995, pp. 158–159), suggesting that even if an attitude was acquired via bypassing, this need not imply that the agent is compelled to possess the attitude.

14 For a similar interpretation, see Pugh (2019, p. 81).

15 These two sets of features are not clearly independent; a change in an agent’s attitudes, for example, may result in a change in her abilities, or the degree to which she has them.

16 I have replaced “input” with “attitude” for ease of presentation, though this seems in line with Fischer’s use. For example, Fischer seems to refer to reasons (p.197), desires, (p. 199) and elements of our mental economy (p. 198) as inputs.

17 Mele suggests that excluding this component concerning prior responsibility would still result in a sufficient condition for not freely *A-*ing (Mele 2019, pp. 136–137).

18 I have replaced “manipulation” with “bypassing.” As Mele makes clear elsewhere, his view is intended to apply to cases in which there is no manipulator present as well (Mele, 2019, pp. 27, 58).

19 Notice that BS would not allow for this possibility, since having these opportunities would not change the way in which the attitude was acquired.

20 A further potentially relevant feature may be that the opportunity he had was relatively brief and insubstantial.

21 This is not a criticism of Fischer. When introducing this notion of opportunity, Fischer is focusing on cases of manipulation that are not hard cases (Fischer, 2012, pp. 203–204).

22 In earlier work, Fischer and Ravizza offered a brief explanation of how this might happen for Judith (Fischer & Ravizza 1998, p. 235). Shaw argues that this version of the view is implausible, since it implies that merely becoming aware of the desire makes the agent free again (Shaw, 2014, pp. 5–6). The version I suggest, and Fischer’s more recent discussion (Fischer, 2012, Chapter 11), avoid this implication.

23 It may be worth noting that what I am suggesting here is a view of when the agent would act freely, which is consistent with claiming that even in these scenarios, Judith may be *less* free with regard to the action than a comparable agent. We will return to degrees of freedom in the next section.

24 There is a further criticism of bypassing views that I do not discuss here; one first put forth by Arpaly (2002, p. 127, 2006, pp. 109–116), and more recently by Cyr (2020, p. 2390) and Shaw (2014, pp. 7–8). The original responses to Arpaly by Mele (2006, pp. 179–184) and Haji and Cuypers (2008, pp. 58–60) apply to more recent versions as well, and Mele repeats this response while replying to Cyr (Mele, 2020, p. 3148).

25 For example, the current California Penal Code concerning the use of anti-libidinal interventions (ALIs) for sex offenders “applies to offenders who have committed one of a number of specified sexual offences against a victim under the age of thirteen. The statute imposes ALIs on eligible offenders as part of their criminal sentence, at the court’s discretion upon an offender’s first commission of one of the relevant offences, and as a matter of mandate upon a second commission of one such offense” (Forsberg, 2018, p. 46).

26 In fact, bypassing can occur even if there was no agent behind the bypassing. For discussion, see Mele (1995, pp. 168–169, 2006, p. 141, 2019, p. 27).

27 However, it is possible that not *all* NNs that help the state achieve its goal will have effects significant enough to undermine freedom for some actions in any individual. It may be possible, for example, that an NN does not produce very significant effects in any one individual, yet when administered to enough subjects, ends up reducing the rate of re-offending. However, assuming that such an NN does not affect everyone equally, it may still be likely to have effects that are significant enough to, at least, mitigate freedom with respect to some actions, in some offenders. This may be even more likely if such an NN is only administered to perpetrators of particularly severe, and/or multiple, offenses. I am grateful to an anonymous reviewer for suggesting that I consider this possibility.

28 I am grateful to an anonymous reviewer for pressing me to consider this objection.

29 I am here supposing that the offer made to the offender is not a coercive one. See Pugh (2018) for discussion.

30 I do not mean to suggest that this is likely; I simply wish to make a conceptual point.

31 It is important to note, however, that I am not suggesting that offering this therapy would *eliminate* the pro tanto reason the state has to refrain from implementing the NN in virtue of the fact that it would initially undermine subjects’ freedom with respect to some actions. It would, however, help to counteract the strengthening of that reason in virtue of undermining that freedom for a long period of time.

32 For discussion of degrees of control, see Shepherd (2014), for degrees of reasons-responsiveness and reasons-sensitivity, see Coates and Swenson (2013) and Kaiserman (2020) respectively, and for a brief discussion of the capacities that might constitute freedom, in relation to degrees, see Clarke (2020, pp. 336–338).

33 For a case that could plausibly be understood in this way, see *One Good Day* (Mele 2019, pp. 29–30, 145–151).

34 For brief discussion, see Mele (2010, pp. 108–109).

35 For a collection of readings on this, see Widerker and McKenna (2003).

36 Vincent also suggests that there will be a tension between the efficacy of the NN as a form of rehabilitation, and the negative impacts the NN might have on offenders, in terms of moral responsibility (Vincent, 2014, p. 36). Although Vincent also appeals to Fischer and Ravizza’s view, she interprets them differently, perhaps as a view like those referred to in fn. 11 above (Vincent, 2014, p. 33).
